# Towards the Properties of Different Biomass-Derived Proteins via Various Extraction Methods

**DOI:** 10.3390/molecules25030488

**Published:** 2020-01-23

**Authors:** Lin Du, Pablo J. Arauzo, Maria Fernanda Meza Zavala, Zebin Cao, Maciej Pawel Olszewski, Andrea Kruse

**Affiliations:** Department of Conversion Technologies of Biobased Resources, Institute of Agricultural Engineering, University of Hohenheim, Garbenstrasse 9, 70599 Stuttgart, Germany; PabloJ.Arauzo@uni-hohenheim.de (P.J.A.); mezazava@gmail.com (M.F.M.Z.); zebin.cao@uni-hohenheim.de (Z.C.); Maciej.Olszewski@uni-hohenheim.de (M.P.O.); Andrea_Kruse@uni-hohenheim.de (A.K.)

**Keywords:** lignocellulosic biomass, protein extraction, subcritical water extraction, protein characterization

## Abstract

This study selected three representative protein-rich biomass—brewer’s spent grain (BSG), pasture grass (PG), and cyanobacteria (*Arthrospira platensis*; AP) for protein extraction with different extraction methods (alkaline treatment, aqueous extraction, and subcritical water extraction). The yield, purity, molecular weight, oil–water interfacial tension, and thermal stability of the obtained proteins derived from different biomass and extraction methods were comprehensively characterized and compared. In the view of protein yield and purity, alkaline treatment was found optimal for BSG (21.4 and 60.2 wt.%, respectively) and AP (55.5 and 68.8 wt.%, respectively). With the decreased oil–water interfacial tension, the proteins from all biomass showed the potential to be emulsifier. BSG and AP protein obtained with chemical treatment presented excellent thermal stability. As a novel method, subcritical water extraction is promising in recovering protein from all three biomass with the comparable yield and purity as alkaline treatment. Furthermore, the hydrolyzed protein with lower molecular weight by subcritical water could promote its functions of foaming and emulsifying.

## 1. Introduction

According to the United Nations, the world’s population is expected to increase to 9.7 billion in 2050, and this requires a 70% increase in food production [1]. It is, therefore urgent to explore alternative methods of sustainable food processing to ensure food security with less environmental impact [2]. Moreover, the current consumption tends to be more plant-based food products rather than animal-based [3]. This led to more intensive utilization of biomass from different sources like agricultural side streams, waste material, and industrial by-products in the food sector [4,5]. Protein is a particularly essential nutrient for human development and health, and rich-protein foods have the potential to decrease worldwide malnutrition [6,7]. In this context, developing sustainable protein refinery techniques from various biomass are required to meet the increase of global protein demand [8].

The high productivity, low cost, and the variety of sources are essential advantages for the food industry acting as structural elements and techno-functional ingredients [9]. Beyond direct consumption for nutritional purposes, proteins are widely used as important ingredients in the food industry [10]. Proteins are macromolecular biopolymers, consisting of amino acid building blocks, and the structure of these building blocks determines protein properties. The properties of the protein that influence its functions in food systems include size, shape, net charge, polarity, structure, composition, and changes with chemical environments. The functionalities of proteins are determined by their structure [11]. For example, the hydrolyzed protein fractions with the increased number of free amino acids and carboxyl groups possess enhanced solubility, digestibility, and technofunctionality [10,12]. The amphiphilic attribute of protein can reduce the surface interfacial tension, which makes them utilized as emulsion and foaming agents [13,14]. Proteins are always obtained in the form of hydrolysate, with the reduced molecular size of protein contributing to the abovementioned functions. In this study, three significant properties (surface tension, molecular size, and thermal stability) are investigated on the extracted proteins.

Lignocellulosic biomass requires pretreatments to release its protein fraction because its recalcitrance depends on its complex composition and factors such as the degree of cellulose polymerization, lignin content, hemicellulose covering, porosity, and fiber strength [15]. Moreover, plant proteins always demonstrate various physical and chemical properties. Taking grass, for instance, the high-lignocellulosic content and cellulosic structure in grass reduce protein extraction yield [10]. The presence of both hydrophilic and lipophilic proteins in grass results in variable protein solubilities in water. The cellulose structure should be disrupted before extraction, and solvent or heating treatment is unavoidable during the extraction. Thus, grass protein is conventionally obtained by pressing or shearing to remove fibers, followed by a heat coagulation process that occurs at high temperatures [16]. Brewer’s spent grain (BSG) known as the residue biomass in the brewing industry and usually contains 10–26 wt.% of protein. The valorization of this industrial by-product can be realized by protein extraction and further conversion [17,18]. Attributing to the disrupted lignocellulosic structure during the beer production, BSG protein is more accessible during extraction. In the last decade, microalgae have been used to produce biofuel and intensively investigated as a new source of functional and nutritious compounds, such as lipid, pigment, and protein. Microalgae contain 40–60 wt.% of protein, which is comparable to that of conventional food protein sources. However, these proteins cannot be digested in full extent, because a complete cell and protein extractability is usually limited by the rigid cell wall [2,19,20]. Furthermore, the types of proteins and their amino acids composition influence the protein accessibility during extraction. Glutelin found as a major protein in BSG, has a high solubility in alkaline solution [21]. A previous study found that the distribution of amino acids indicates protein solubility; non-essential amino acids tend to be more insoluble [22]. BSG contains the highest non-essential amino acids content among the studied feedstock materials (Table S1). Leafy protein Rubisco is usually obtained at pH lower than 10 [23]. The neutral or alkaline condition is not preferred for its recovery. However, the isoelectric point of over 50% plant protein is in the acidic range, and the proteins demonstrate high solubility at alkaline condition. It is, therefore, of great importance to select suitable extraction methods, according to different protein characteristics.

The high productivity, low cost, and wide variety are important advantages for biomass acting as protein feedstock [9]. The primary challenge of protein extraction from waste material or industrial by-products is to avoid denaturing or affecting their functional properties [24]. Pretreatment methods are broadly classified into biological, physical, chemical, and combinatorial [15,25]. All methods have advantages and disadvantages, while gentle treatments may not release all protein from the cell obtaining highly viscous extracts; harsh treatments can reduce viscosity but may result in the inactivation of labile proteins [26]. An effective pretreatment method should be simple, cost-effective, and most importantly, it should safeguard the fraction of interest by avoiding considerable losses [25]. Alkaline treatment is the most common method applied to agricultural and food residues for protein extraction. Through disulfide cross-linking breakage, the extractability of the protein is enhanced. It consists of the disruption of cell walls to extract protein easier and this extractability is influenced by certain extraction conditions such as biomass type, pH, temperature, and extraction time [10,27,28]. Subcritical water treatment is emerging as a “green” extraction method avoiding the introduction of chemicals. With a decreased density and dielectric constant at subcritical conditions, water becomes a better solvent for protein. The increased ion concentration provides biomass the environment of hydrolyzing without the introduction of acid [29,30,31]. However, proteins are reported denatured in some studies due to high extraction temperature and pressure.

From the aspect of valorization of waste biomass and sustainable refinery, the present study aimed to reveal the optimal protein extraction method for biomasses with different compositions. Brewer’s spent grain (BSG), pasture grass (PG), and the cyanobacteria *Arthrospira platensis* (AP) were selected as a representative of lignocellulosic biomass with varied composition. As a comparison to aqueous extraction, alkaline treatment and subcritical water extraction were applied. The properties (molecular weight, oil–water interfacial tension, and thermal stability) that can affect functionality were investigated on the obtained protein concentrates.

## 2. Results and Discussion

### 2.1. Extraction Method Comparison

In this study, protein extraction yield involved two aspects, protein extraction into the aqueous phase and precipitation. The latter was performed identically for each biomass. The extraction parameters are critical for high quality and cost-effective protein production. Alkaline treatment and aqueous extraction are relatively mild, considering extraction temperature (40 °C). However, introducing alkaline solution will increase production cost, and the extraction yield depends mainly on the volume of alkaline solution [32,33,34]. Subcritical water extraction is regarded as a ‘green’ method with shorter extraction duration (20 min in this study) [29,35]. However, the harsh reaction condition (200 °C, 40 bar) may cause protein degradation and denaturation [36]. The effects of extraction parameters on the protein properties will be discussed in the following.

Aqueous extraction acted as a reference in this study, as well. It could be concluded that with chemical (alkaline treatment) and hydrothermal (subcritical water extraction) treatment, the protein extracted yields of all three biomass were improved. During aqueous extraction, the osmosis-caused diffusion in water through cell or cellulose structure was not strong enough for protein molecule permeation [37]. Strong alkaline conditions can partly remove the cellulosic structures with soluble protein dissolving in water [27,28,38]. Alkaline treatment showed the highest protein extraction yield for BSG (21.4 wt.%) and AP (55.5 wt.%), which is comparable with previous studies [39,40].

For PG, however, large shares of leaf proteins are located in the plasma membrane or cytoplasm. The mechanical pressing is always applied to fresh grass biomass to squeeze the protein-rich green juice in grass biorefinery [16,41]. Therefore, a combination of the cellulosic matrix disruption and mechanical fragmentation is necessary to promote the grass protein recovery. Subcritical water treatment increased by more than 5% of protein extraction yield comparing with alkaline treatment. It resulted from cellulose hydrolysis and cell wall disruption in subcritical water [42]. Nevertheless, the yield (6.7 wt.%) was far less than the previous study on protein extraction from tea leaves, with the yield 95 wt.% at 95 °C during 4 h extraction [34]. Extraction time was found crucial factor for an increased extraction yield [43].

The protein precipitation yield is highly interfered by the separation method. Many reported that the TCA precipitation reduces the solubility of concentrated protein [44]. Moreover, TCA is ineffective in precipitating unstructured or disordered protein that composes 30% of eukaryotic and 4.2% of eubacterial protein [45]. Thus, TCA precipitation is a preferred method for AP protein separation after alkaline treatment with a total extraction yield of 55.5 wt.%. After treated with subcritical water, the hydrolyzed protein/peptides fractions were not efficiently recovered with an overall yield of 19.9 wt.%. Nevertheless, by removing salts and proteases, the high protein purity can be obtained by TCA precipitation. It is regarded as the most efficient protein separation method regardless of protein source [46,47,48].

### 2.2. Protein Extraction Yield and Purity

**Figure 1:**
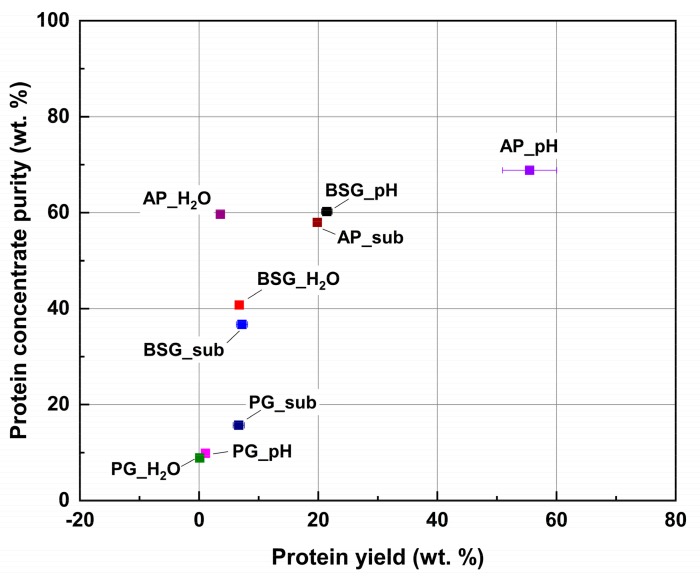
Graphical integration of protein extraction yield and protein concentrate purity.

Neither the protein extraction yield nor the protein concentrate purity can solely indicate the protein recovery yield. The integration of both aspects was shown in Figure 1. Alkaline treatment followed by TCA precipitation is the optimized method for protein recovery from AP and BSG. Meanwhile, subcritical water treatment is more suitable for PG protein. It should be noted that although subcritical water treatment of AP and BSG showed lower purity than aqueous extraction, the yields were much higher. The lower purity resulted from carbohydrates contamination that was extracted during subcritical treatment. Other water-soluble substances such as sugars and salts were found extracted during the alkaline treatment [43]. Although TCA precipitation is the most promising method to separate protein, the contamination of polysaccharides and DNA is not avoidable [49]. All in all, given recovery yield, alkaline treatment, and subcritical water extraction were preferable for the three biomass. To conclude the ideal protein recovery method for specific biomass, a comprehensive economic analysis regarding capital and energy input, environmental consideration, as well as the protein quality and the market price is needed [33].

The studied extraction methods accord to the concept of green chemistry that hazardous substances were neither used nor generated [50]. With subcritical water treatment, chemicals are completely avoided, and the extraction time was shortened by over 83% (Table 1). Additionally, the application of a continuous process can reduce energy input compared with batch extraction [51].

In the conclusion, one possible integration of protein extraction from biomass in industrial applications is introduced, with alkaline and subcritical water treatment. Although alkaline treatment led to high protein extraction yield, unextracted protein remained in the residue. The previous study showed that the protein residue of BSG, after alkaline treatment, contains 3.63% of N [28]. Further subcritical water treatment might further destroy the lignocellulosic structure of the residues, resulting in a promoted protein extraction efficiency. The combination of the two “green” extraction methods is a potential optimization approach in industrial protein biorefinery.

Nevertheless, further investigation should focus on the extraction mechanism of protein from the alkaline lignin cellulose residue under the subcritical condition. In the context of sustainability, the by-products can be applied and reused to reduce the waste. For example, the lignocellulose-rich solid residue might be used for animal feeding, energy-rich material production, and fermentation of ethanol. The aqueous waste that is rich in organic content, has the potential for biogas production.

### 2.3. Molecular Weight

**Figure 2:**
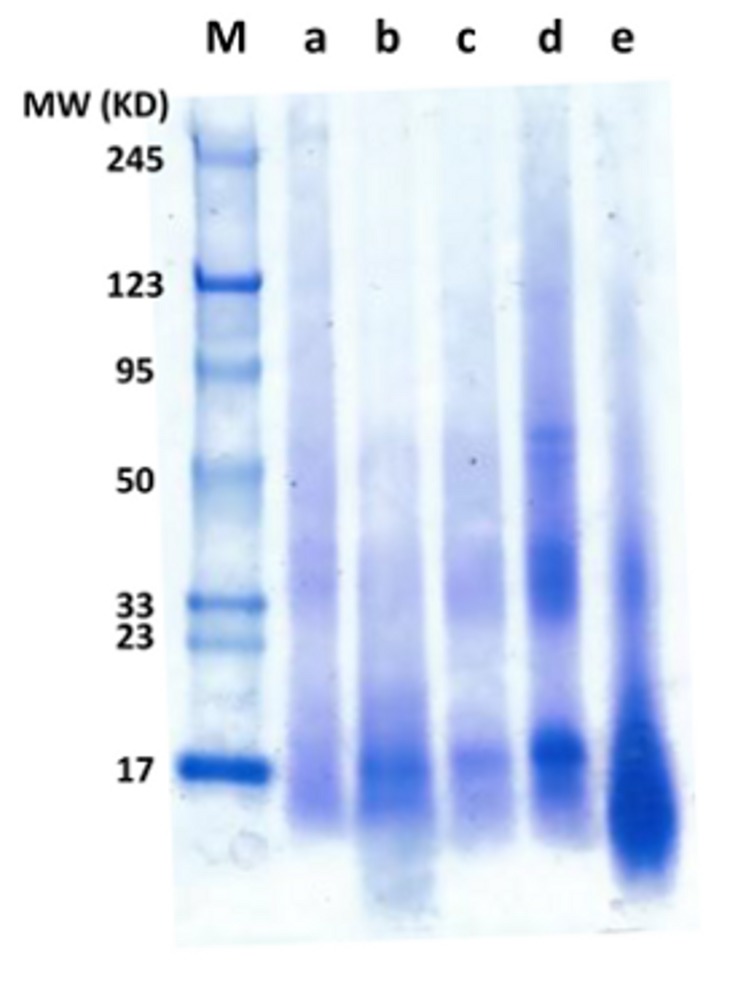
The molecular weight distribution of protein concentrates (protein concentration 0.5 *w*/*w*%). M: marker; a: BSG_pH; b: PG_pH; c: AP_pH; d: AP_H_2_O; e: AP_sub.

The hydrolyzed protein fractions with lower molecular weight are preferred in the food industry because of improved nutritional quality and functional properties. The properties include solubility, digestibility, viscosity, emulsification, and gelation [52]. The molecular weights of obtained protein concentrate from three biomasses with different pretreatment methods varied (Figure 2). BSG_pH and AP_H_2_O covered the full range of molecular weights that were marked. As was discussed in the previous study, the BSG protein concentrates recovered with pH shifting method is promising as functioning agents in the food industry [28]. The BSG protein fractions of molecular weights between 14.5 and 50 KD perform better given emulsifying and foaming [39,53].

The molecular weight of PG_pH and AP_pH mainly located in the range lower than 50 KD. During alkaline treatment, the solubility of protein was improved as a result of the disruption of the disulfide crosslink. Meanwhile, peptides fractions were generated. Besides, increased temperature and pH can cause protein denaturation and hydrolysis [10]. The structure unfolding was also observed on the AP_pH protein when comparing with AP_H_2_O.

The more intensive dye of AP_sub in the lower molecular weight zones revealed mainly protein hydrolysates existing in the extract. The previous study of the algal protein showed better emulsifying properties on the acidic hydrolysates, which had similar molecular weights distribution as in our study [12]. Meanwhile, the solubility of protein concentrate was enhanced via subcritical water treatment. Excellent foaming properties were found with protein concentrates after the severe extraction process [54,55]. Hence, subcritical water treatment provides a novel alternative for biomass protein extraction with promoted functionalities.

### 2.4. Surface Tension

**Figure 3:**
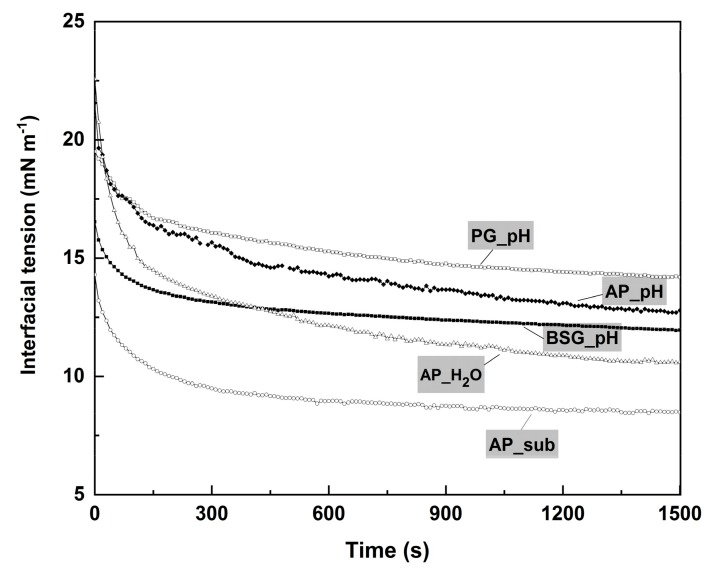
Oil−water interfacial tension profiles of protein concentrate (protein concentration = 0.1 wt.%). Relative standard deviations are less than 5% (*n* = 3).

To investigate the potential of obtained protein concentrates as an emulsifier, the oil–water intersurface tension (γ) of five different protein concentrates were measured along dropping time (30 min). Firstly, all the protein concentrates were able to decrease the interface tension. It generally indicated the presence of surface-active proteins in the extracted concentrates [56,57]. Figure 3 displayed the lowest final interfacial tension of AP protein concentrates obtained with subcritical water treatment. The decreased speed and decreased extent of interfacial tension were comparable with that of a small-molecular surfactant in the previous study [58]. The capacity and the rate of decreasing interfacial tension of AP_sub indicated its excellent potential as a food emulsifier. It should be noted that other compounds with a low molecular weight such as carbohydrates and lipids could stabilize emulsion on the oil–water intersurface [13,58]. These impurities that were observed in extracted protein contribute to emulsion stabilization as well.

### 2.5. Thermal Stability

**Figure 4:**
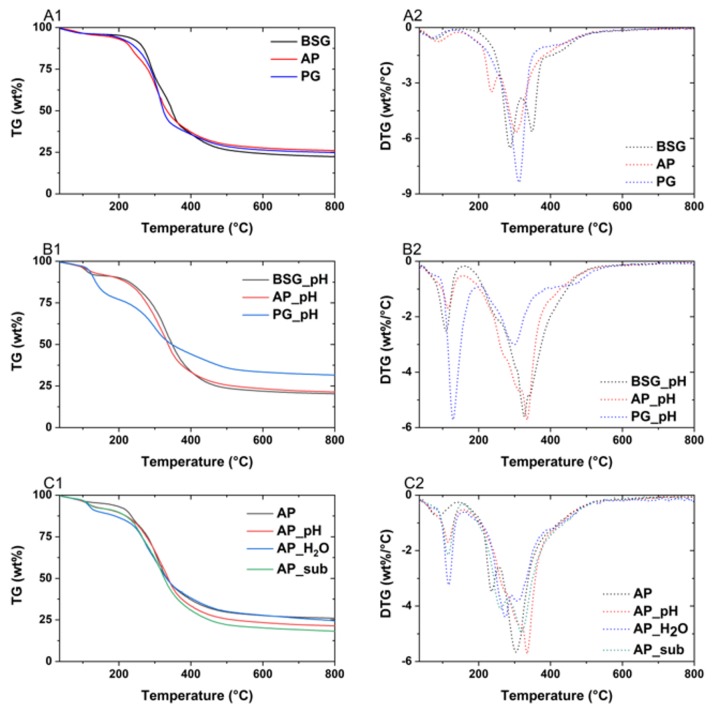
Comparison among TGA of original feedstock (**A**), proteins isolated from different feedstock by alkaline treatment (**B**), and *Arthrospira platensis* (AP) protein extracted by different treatments (**C**).

This study exploited the thermogravimetric analysis (TGA) to explore the thermal behavior of the original feedstock and the extracted proteins by different methods. Graphs in Figure 4A indicated the variety of thermal behavior of different feedstock. Two peaks present in the derivative ther mogravimetric (DTG) curves of BSG and AP, while PG shows only one major peak. The peak in the range of 250–300 °C is associated with hemicellulose decomposition. Hemicellulose is mainly composed of carbohydrates [59]. BSG contains more hemicellulose [60] than AP and PG as the remaining carbohydrates from brewing. The second peak ranges from 300 to 400 °C, which was presented in all three feedstocks, was related to the cellulose decomposition. It can be found that PG is mainly composed of cellulose because the leafy tissues are composed of three lamellas parts with cellulose inside [61]. The less stability of AP can be explained by the high content of protein, which reacts with carbohydrates via Maillard reaction pathways at 150 °C [62] and decompose to amino acids at a lower temperature.

The thermal behavior of the extracted proteins is shown in Figure 4B,C. Figure 4B compared the proteins from different feedstock by alkaline treatment, while Figure 4C focuses on AP proteins isolated via various treatments. The first peak observed at the temperature range between 100 and 150 °C was assigned to dehydration. The PG isolated protein (alkaline treatment) was most hydrophilic, followed by BSG and AP (Figure 4B2). Figure 4C shows that AP lost the least mass during this temperature range, which further proved the hydrophilicity of the isolated proteins. In terms of the proteins, the hydrophilicity trend was AP_H_2_O > AP_sub > AP_pH. The second peak was observed at the temperature range between 250 and 350 °C and it was associated with the start of thermal decomposition of protein (volatilization of the proteins). A positive correlation between the purity of the isolated protein and the thermal stability was found by Ricci et al. (2018), and this is clearly indicated in Figure 4B2. AP_pH, with the highest purity, had the highest peak temperature. On the contrary, the peak temperature (second step) of PG_pH was 20 °C less than that of AP-pH due to the lowest purity. Due to the similar purity, AP_pH and BSG_pH had the almost same peak temperature. In Figure 4C2, even the purities of AP_H_2_O and AP_sub are almost the same; the peak temperature of AP_H_2_O was much lower than that of AP_sub. It might be that the isolated protein by aqueous treatment contained a high amount of impurities, which resulted in the instability of the AP_H_2_O protein. Other minor peaks presented in the DTG curves are the degradation of the non-protein substances [63].

It can be concluded that alkaline treatment is only suitable for extracting proteins from BSG and AP due to the high purity and thermal stability. The subcritical water extraction is also a promising method to obtain proteins from AP due to the relatively high thermal stability, and fewer chemicals are needed for this method.

## 3. Materials and Methods

### 3.1. Materials

Brewer’s spent grain (BSG): the brewer’s spent grains with a moisture content of 78 wt.% were obtained from the Hoepfner Brewery factory (Karlsruhe, Germany). It was stored at −15 °C until processed, to avoid the microbiological activity that takes place.

Pasture grass (PG): the pasture grass was obtained freshly from a farm in Münzesheim Kraichtal (Karlsruhe, Germany), and cut into pieces with an average length of 2–3 cm. It was stored at −15 °C for further treatment.

*Arthrospira platensis* (AP): *Arthrospira platensis* was obtained from IGV GmbH (Nuthetal Germany) in dried pellet form with a dry weight of 94.9 wt.%. Before extraction, *A. platensis* pellets were milled with Cyromill (Retsch GmbH, Haan, Germany) at frequency 30 s^−1^ for 1 min to a homogenous fine powder.

The compositions of the studied feedstock materials are shown in Table 2.

### 3.2. Protein Concentrates Preparation

#### 3.2.1. Alkaline Treatment

BSG, PG, and AP were suspended in 0.1 M NaOH and distilled H_2_O to reach a pH > 11 with a final solid to liquid ratio of 1:10 (*w*/*w*). The mixture was stirred for 2 h at 40 °C and then centrifuged at 13,500× *g*, 4 °C for 20 min (Z 326 K, HERMLE Labortechnik GmbH, Wehingen, Germany). The protein-rich supernatant was taken for precipitation and further analysis.

#### 3.2.2. Aqueous Extraction

The same amount of feedstock BSG, PG, and AP were mixed with distilled water (solid to liquid ratio 1:10 *w*/*w*). Extraction took place at 40 °C for 2 h and the same separation method as for alkaline treatment was applied.

#### 3.2.3. Subcritical Water Extraction

Subcritical water extraction was performed in a semi-continuous reactor at the condition: temperature 200 °C, pressure 40 bar, and flowrate 6 mL/min. After 20 min. extraction duration, corresponding aqueous extracts were obtained and kept for precipitation and analysis.

#### 3.2.4. Trichloroacetic Acid (TCA) Precipitation

The protein-rich aqueous extracts were adjusted to pH 3.0 with 1.0 M trichloroacetic acid (TCA). This pH was reported as the isoelectric points of most plant proteins, at which the interaction between protein molecules and water is minimized, resulting in low solubility [27]. Subsequently, the insoluble protein precipitated and recovered after centrifugation at 13,500 × *g*, 4 °C for 20 min. The obtained pellets were frozen at −15 °C until they were lyophilized in a freeze dryer (ALPHA 1-2LD plus from CHRIST GmbH, Osterode am Harz, Germany).

**Figure 5:**
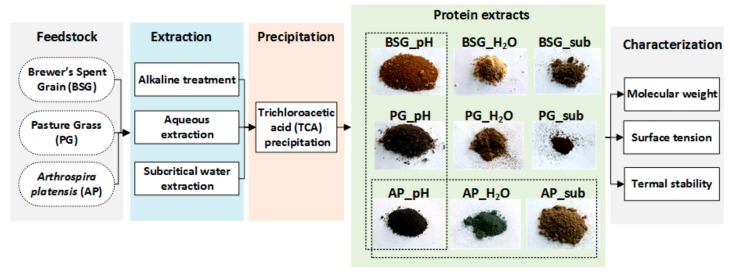
Scheme of the experimental process for protein extraction by three different methods (alkaline treatment, aqueous extraction, and subcritical water extraction) of three types of biomass (brewer’s spent grains (BSG), pasture grass (PG), and *Arthrospira platensis* (AP)).

The freeze-dried extracts are denoted respectively as BSG_pH, PG_pH, and AP_pH (from alkaline treatment), BSG_H_2_O, PG_H_2_O, and AP_H2O (from aqueous extraction), and BSG_sub, PG_sub, and AP_sub (from subcritical water extraction). Figure 5 demonstrates the experimental process and the appearances of the extracted proteins. 

### 3.3. Composition Analysis

The elemental analysis was carried out in an Elemental Analyzer EA3000 Series (EuroVector Instruments & Software Srl, Pavia, Italy) equipped with a thermal conductivity detector (TCD) to determine the percentage composition of CHNS (carbon, hydrogen, nitrogen, and sulfur). Proximate analysis was performed according to the standard test method ASTM D1762-84 (2013) to determine moisture content and ash content in solid samples. The elemental analysis was applied to all the feedstock materials and protein isolates, and a proximate analysis was applied only to the feedstock.

Concerning the low amounts of protein extracts obtained, it is challenging to perform Kjeldahl protein or amino acid analysis. The protein content of the extracts was estimated with the total nitrogen content, and the nitrogen to protein factor 6.25 [64] (Equation (1)).

(1) $$\mathrm{Protein}\;\mathrm{content}\;\left(\mathrm{wt}.\%\right)=\;\mathrm{Nitrogen}\;\left(\mathrm{wt}.\%\right)\times \;6.25.$$

### 3.4. Lowry Protein Determination

The protein in aqueous extracts was treated with copper (II) salt under alkaline conditions and then reacted with Folin and Ciocalteu′s phenol reagent, which generated absorption at 750 nm (Gerhardt 1994; Lowry et al., 1951). The concentrations of protein in the extracts were determined according to the standard. Reagents A (2.0% Na_2_CO_3_ + 0.1 N NaOH), B (0.5% CuSO_4_ with 1% C_4_H_4_NaO_6_ 4H_2_O), and C (50 mL A + 1 mL B) were prepared as in the previous study. Chemicals were purchased in analysis pure grade from Merck KGaA (Darmstadt, Germany) and VWR International GmbH (Darmstadt, Germany). Bovine serum albumin (purity ≥ 98.5%, Merck KGaA) was used as a standard with concentration from 20 to 200 mg/L. The test was performed in a 96-well microplate. Of the standard or sample mixed 40 µL with 200 µL of reagent C reacted for 10 min. Then 20 µL of the 1 N Folin and Ciocalteu′s phenol reagent (VWR International GmbH) was added and mixed well. The mixture solutions were kept in the dark for 60 min before the absorbance was read with 750 nm in an EPOCH2 plate reader (BioTek Instruments GmbH, Bad Friedrichshall, Germany).

### 3.5. Extraction Yield and Purity Determination

The protein extraction yield and purity were determined based on Equations (2) and (3), respectively.

(2) $$\mathrm{Protein}\;\mathrm{extraction}\;\mathrm{yield}\;\left(\mathrm{wt}.\;\%\right)\;=\;\frac{\mathrm{Amount}\;\mathrm{of}\;\mathrm{dried}\;\mathrm{protein}\;\mathrm{precipitate}\;\left(\mathrm{mg}\right)}{\mathrm{Protein}\;\mathrm{content}\;\mathrm{in}\;\mathrm{biomass}\;\left(\mathrm{mg}\right)}\times \;100\%.$$

(3) $$\mathrm{Protein}\;\mathrm{purity}\;\left(\mathrm{wt}.\%\right)=\;\frac{\mathrm{Protein}\;\mathrm{content}\;\mathrm{in}\;\mathrm{precipitate}\;\left(\mathrm{mg}\right)}{\;\mathrm{Protein}\;\mathrm{preciAmount}\;\mathrm{of}\;\mathrm{dried}\;\mathrm{protein}\;\mathrm{precipitate}\;\left(\mathrm{mg}\right)}\;\times 100\%.$$

### 3.6. Protein Functional Properties Characterization

Due to the higher protein recovery yield and research interest, the functionality characterization was performed on the extracts: BSG_pH, PG_pH, AP_pH, AP_H_2_O, and AP_sub.

#### 3.6.1. SDS-Polyacrylamide Gel Electrophoresis (SDS-PAGE)

SDS-PAGE carried out on the protein extracts to observe the molecular weight with the Mini-protean II system (Bio-Rad Laboratories GmbH, München, Germany). The solutions with protein concentrations of 0.5 wt.% were prepared with distilled water for BSG_pH, PG_pH, AP_pH, AP_H_2_O, and AP_sub. The suspensions were diluted with buffer solution containing 5% ß-mercaptoethanol (purity ≥ 98%), 0.5 M Tris-HCl pH 6.8 (purity ≥ 99.8%), 25% glycerol (purity ≥ 99%), 10% SDS, and 5% bromophenol blue indicator (Merck KGaA). Of each sample 10 µL was loaded into each cell and followed by the electrophoresis at 200 V for 40 min. The gel was stained with Coomassie brilliant blue R-250 (Bio-Rad Laboratories GmbH, München, Germany) for 2 h and washed with 15% methanol and 10% acetic acid solution overnight. A pre-stained marker (Roti^®^-Mark, Carl Roth GmbH + Co. KG, Karlsruhe, Germany) with a range from 17 to 245 kDa was used to determine the molecular weight distribution.

#### 3.6.2. Interfacial Tension

The interfacial tension was carried out on five protein extracts with a concentration of 0.1 wt.%. A drop-shape analyzer (DSA-G10, MK2, Krüss, Charlotte, NC, U.S.A.) was used to analyze the interfacial tension at the oil−water interface. A drop of the sample was formed at the tip of the syringe, whose shape was calculated from the balance of force on the drop following the Young−Laplace equation. The interfacial tension was then determined from the shape of the drop. Sample solutions were firstly put in a syringe with a narrow tip (d = 0.90 mm) then into a cuvette filled with the oil phase (MCT, Miglyol 812, Cremer Oleo GmbH & Co. KG, Hamburg, Germany). The cuvette was placed onto the optical bench where the light source can go through. The shape profile of the drop was recorded by a camera and processed by the software. The densities of samples were measured by a digital density meter (DMA 35N, Anton Paar Physica, Ostfildern- Scharnhausen, Germany).

#### 3.6.3. Thermogravimetric Analysis

The thermogravimetric analysis (Netzsch STA 449 F5, NETZSCH-Gerätebau GmbH, Selb, Germany) was conducted to analyze the thermal stability of the original feedstock and isolated proteins. The sample was loaded into an Al_2_O_3_ crucible and heated to 800 °C with a constant heating rate of 10 °C/min in the N_2_ atmosphere (70 mL/min).

Extractions and analyses were implemented at least three times, and the relative standard deviation of each result was smaller than 10%. The thermogravimetric analysis was conducted only once. The main error of the experiments came from biomass heterogeneity and temperature control.

## 4. Conclusions

The study demonstrated the protein recovery efficiency from various biomasses. Alkaline treatment is suitable for BSG and AP with a high protein yield and purity, while subcritical water extraction favorable for PG. The extracted protein demonstrated superior or comparable properties in comparison with original biomass and other common proteins. The hydrolyzed product from subcritical water extraction contributes to the emulsification function, which suggests it a promising sustainable extraction technique.

**Figure 6:**
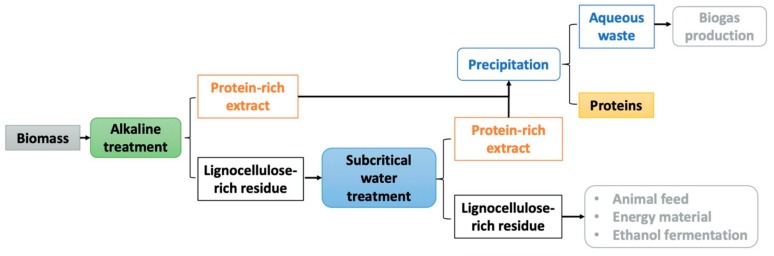
Integration of alkaline and subcritical water treatment to optimize protein extraction from biomass.

This can be used as the basis for a biorefinery. First an alkaline treatment is applied. This leads to a protein-rich extract and a lignocellulose-rich residue (Figure 6). This residue is extracted with hot liquid water (subcritical water). Here again a protein rich extract was generated, which is mixed with the extract produced by the first, alkaline extraction. From this mixture of aqueous liquids, solid protein is precipitated. This is the wished product. The aqueous solution, left after protein precipitation is delivered to a biogas plant. The solid lignocellulose-rich residue, twice extracted, is still reach in cellulose. It can be further processed, e.g., by splitting of cellulose to glucose and consecutive ethanol production (2nd generation bioethanol), or it can be pyrolyzed to get a solid fuel.

Such a double-extraction could be for example integrated in a large brewery, in this case BSG is the feedstock. Here, the extraction could be integrated in the steam-heating system and the solid residue may be used to produce the heat.

In the case of grass, it is interesting to produce proteins from grass directly, instead to use it as fodder for cattle production. The efficiency was higher and the greenhouse gas production was much lower. In this case it has to be investigated to use the solid lignocellulose-residue e.g., for paper production, because of the fiber-like structure.

Today, more and more people want to avoid protein from animals, because of different reasons. To produce proteins from plants will become more and more important and would be an interesting business case.

## Figures and Tables

**Table 1:** A comparison between three extraction methods conditions and the obtained protein yield and purity.

		Alkaline Treatment	Aqueous Extraction	Subcritical Water Extraction
Extraction time/duration (min.)		120	120	20
Temperature (°C)		40	40	200
pH		11	7	7
Solvent involved		0.1 M NaOH	Water	Water
Protein yield (wt.%)	BSG	21.4 (0.9)	6.8 (0.1)	7.2 (0.9)
	PG	1.1 (0.1)	0.1 ^a^	6.7 (0.9)
	AP	55.5 (4.6)	3.6 (0.1)	19.9 (0.4)
Protein concentrate purity (wt.%)	BSG	60.2 (0.7)	40.7 (0.3)	36.7 (0.1)
	PG	9.8 ^a^	8.9 (0.3)	15.7 ^a^
	AP	68.8 (0.2)	59.7 (0.1)	58.0 (0.3)

Values are expressed as mean (*n* = 3). In parentheses: standard deviation. ^a^ Standard deviation is less than 0.05.

**Table 2:** The composition of the three feedstock materials.

Composition (wt.%)	BSG	PG	AP
Protein ^a^	21.9	9.14	55.9
C	50.9 (0.3)	44.8 (0.1)	45.2 ^c^
H	7.0 (0.1)	6.3 ^c^	6.9 ^c^
N	4.3 (0.2)	1.8 ^c^	9.4 ^c^
S	0.2 ^c^	0.1 ^c^	0.6 ^c^
O ^b^	33.6 (0.4)	40.1 (0.1)	32.4 (0.1)
Ash	3.9 ^c^	7.0 (0.1)	5.5 (0.1)
Dry Weight	22 ^c^	29.7 (0.8)	94.9 (0.2)

Values are expressed as mean (*n* = 3). In parentheses: standard deviation. ^a^ Protein content based on the amino acids composition (*n* = 1); ^b^ O content calculated according to elementary and ash composition; ^c^ Standard deviation is less than 0.05.

## Supplementary Materials

The following are available online. Table S1: The amino acids composition of the studied feedstock.
