# Supplementary material for: Towards the Properties of Different Biomass-Derived Proteins via Various Extraction Methods
Source: Molecules. 2020 Jan 23;25(3):488. doi: 10.3390/molecules25030488 (PMC7037764; doi:10.3390/molecules25030488)
Supplement: Supplementary file 1 [file molecules-25-00488-s001.pdf]

Table S1. The amino acids composition of the studied feedstock

**Amino acids [wt.%]**	**PG**	**BSG**	**AP**
**Asp**	10.24	7.91	11.07
**Thr**	5.35	3.98	4.40
**Ser**	5.01	4.71	4.49
**Glu**	13.03	20.68	19.17
**Gly**	6.57	4.62	4.80
**Ala**	8.13	5.72	7.97
**Cys**	0.89	2.15	0.71
**Val**	6.46	6.08	6.70
**Met**	2.00	1.97	1.88
**Ile**	4.90	4.35	5.56
**Leu**	9.35	8.33	8.46
**Try**	3.23	3.06	3.35
**Phe**	6.12	5.76	4.13
**His**	2.12	2.20	1.38
**Lys**	3.90	3.66	4.26
**Arg**	5.46	5.44	8.28
**Pro**	7.24	9.38	3.39
**Essential**	45.66	41.77	45.05
